# Supplementary material for: Microalgae Inoculation Significantly Shapes the Structure, Alters the Assembly Process, and Enhances the Stability of Bacterial Communities in Shrimp-Rearing Water
Source: Biology (Basel). 2024 Jan 19;13(1):54. doi: 10.3390/biology13010054 (PMC10813777; doi:10.3390/biology13010054)
Supplement: Supplementary file 1 [file biology-13-00054-s001.zip › supplementary figure.pdf]

Article

Microalgae inoculation significantly shapes the structure, alters the assembly process, and enhances the stability of bacterial communities in shrimp rearing water

Chen Lian [1], Jie Xiang [1], Huifeng Cai [2], Jiangdong Ke [1], Heng Ni [1], Jinyong Zhu [1], Zhongming Zheng [1], Kaihong Lu [1] and Wen Yang [1,*]

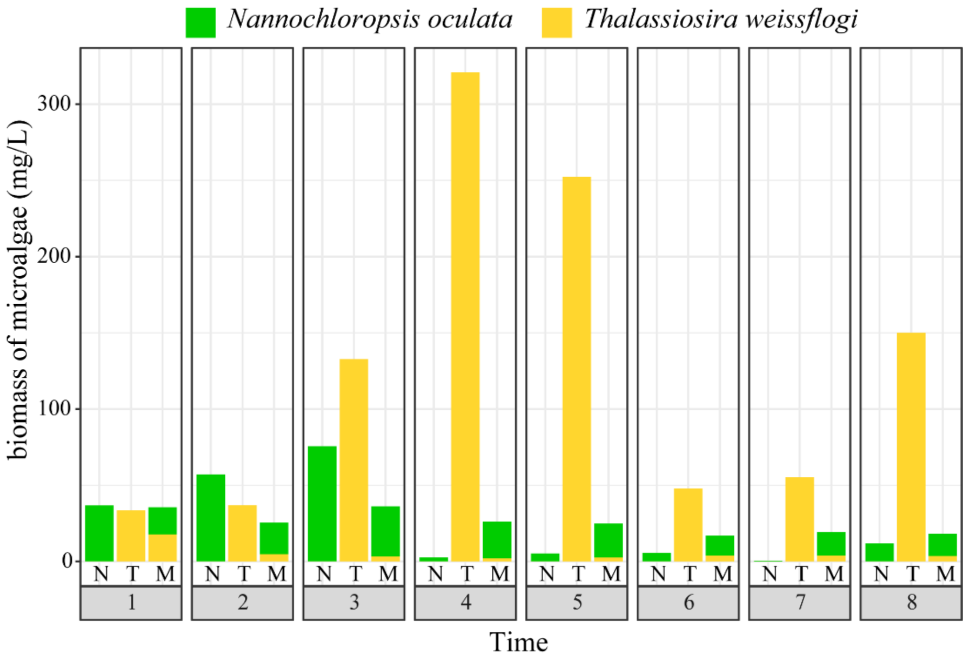

Figure S1. Biomass of different groups of microalgae at different sampling times.

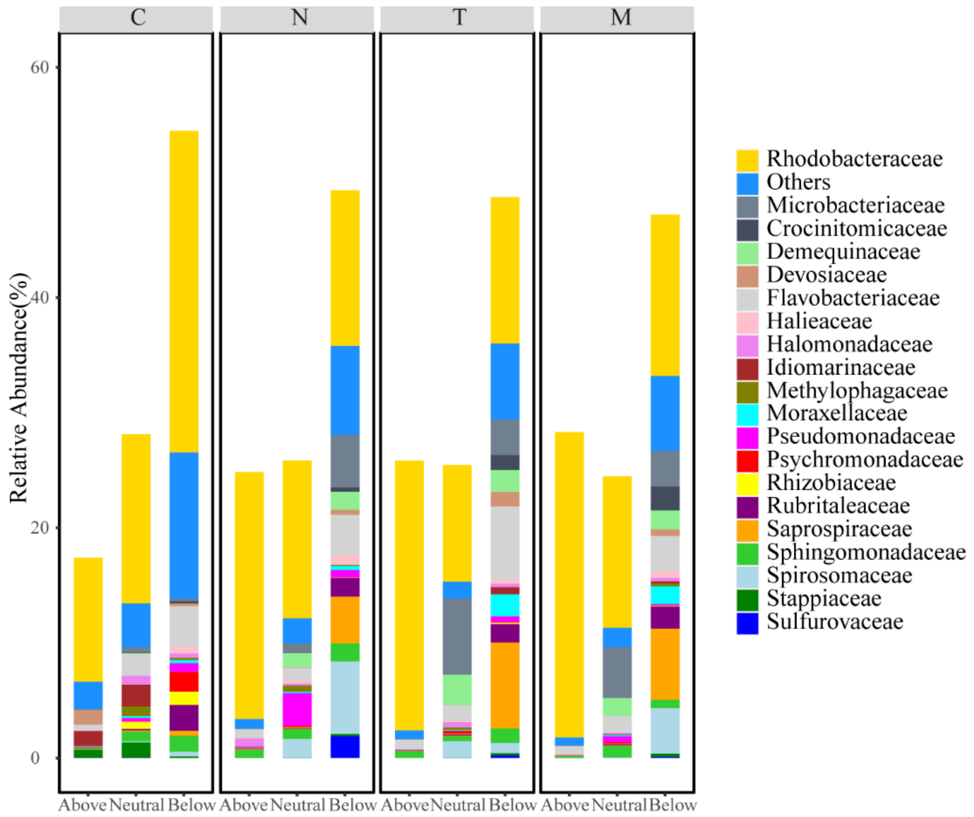

Figure S2. The bar chart (B) shows the taxonomic distribution (family level) of ZOTUs in three parts (above prediction, below prediction, and neutral distribution) predicted by the four sets of neutral models. Above, Neutral, and Below represent above-predicted, under-predicted, and neutral distributions in the neutral model.
